# Large-scale microfluidic gradient arrays reveal axon guidance behaviors in hippocampal neurons

**DOI:** 10.1038/micronano.2017.3

**Published:** 2017-05-08

**Authors:** Nirveek Bhattacharjee, Albert Folch

**Affiliations:** 1 Department of Bioengineering, University of Washington, 3720 15th Ave NE, Foege N423-A, Seattle, WA 98195, USA

**Keywords:** axon guidance, microfluidic gradient, microjet, netrin-1, primary mammalian neuron

## Abstract

High-throughput quantitative approaches to study axon growth behaviors have remained a challenge. We have developed a 1024-chamber microfluidic gradient generator array that enables large-scale investigations of axon guidance and growth dynamics from individual primary mammalian neurons, which are exposed to gradients of diffusible molecules. Our microfluidic method (a) generates statistically rich data sets, (b) produces a stable, reproducible gradient with negligible shear stresses on the culture surface, (c) is amenable to the long-term culture of primary neurons without any unconventional protocol, and (d) eliminates the confounding influence of cell-secreted factors. Using this platform, we demonstrate that hippocampal axon guidance in response to a netrin-1 gradient is concentration-dependent—attractive at higher concentrations and repulsive at lower concentrations. We also show that the turning of the growth cone depends on the angle of incidence of the gradient. Our study highlights the potential of microfluidic devices in producing large amounts of data from morphogen and chemokine gradients that play essential roles not only in axonal navigation but also in stem cell differentiation, cell migration, and immune response.

## Introduction

**Figure 1:**
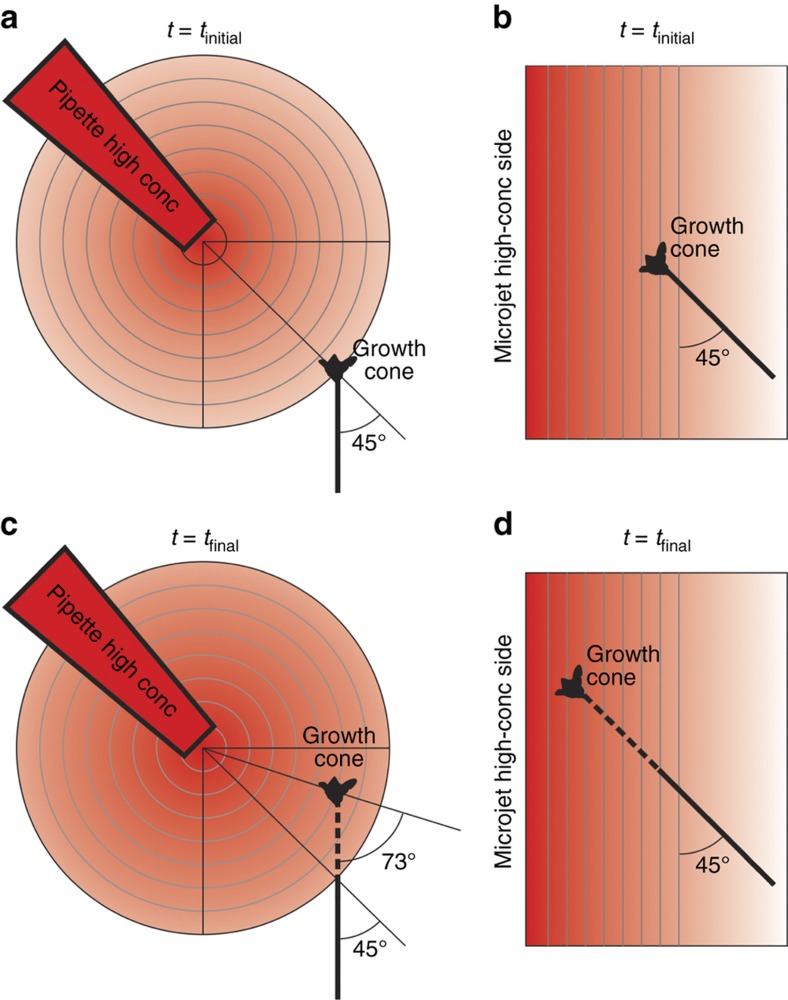
Measurement limitations imposed by the relationship between the axon’s growth path and the gradient’s topology. All the schematics depict a growing neuron that does not turn in the presence of a signaling gradient before growth begins (**a** and **b**) and after the growth measurement is taken (**c** and **d**). We consider two types of gradients: (**a** and **c**) radial concentration isolines in a micropipette-generated gradient (traditional assay), and (**b** and **d**) parallel concentration isolines in the microfluidic gradient generated in our device. Notably, in (**d**), the angle of incidence and the slope of the gradient remain unchanged at a later time-point if the growth cone advances straight, without any deviation, when the concentration isolines are parallel. On the other hand, in (**c**) the angle of incidence and the slope of the gradient change significantly at a later time-point, even when the growth cone advances straight without any deviation, which makes quantitative interpretation difficult (especially for responding neurons). Micropipette-generated gradients evolve over time because, unlike in our microjets device, there is no constant sink for the diffusible molecule.

The challenges associated with investigating the spatiotemporally complex processes of axon guidance *in vivo* have long motivated the development of *in vitro* experimental paradigms, with both explant cultures^1^ and dissociated primary neurons^2^. However, the explant culture system is only suitable for looking at responses of axon populations, and the standard “growth-cone turning assay”—using pulsatile ejection from micropipettes^3^—creates gradients that are constantly evolving and cannot be accurately quantified (as they are dependent on many parameters, such as the exact shape and distance of the pipette, the molecular weight and charge of the chemical, the pulsing frequency and duration, and so on)^4^. Over the last decade, microfluidics has emerged as an attractive technology for interrogating cultured cells with precisely controlled, deterministic, and stable concentration gradients (whether steady-state or time-varying) of biochemical cues^5,6^. In micropipette studies, the gradient originates approximately from a point source, thereby generating radial concentration isolines (Figure 1a), whereas in most microfluidic devices, the concentration isolines are parallel (Figure 1b). In micropipette assays, a hypothetical advancing growth cone encounters an evolving gradient and is exposed to the gradient at varying angles as the growth progresses (Figure 1c), which makes analysis difficult. On the other hand, in a microfluidic device, the same advancing growth cone faces a stable concentration field without changing the angle of incidence of the gradient (Figure 1d). Gradients of biochemical molecules generated by microfluidic devices have been used extensively to study cell guidance, including neutrophil migration^7–9^, breast cancer cell metastases^10^, bacterial chemotaxis^11^, stem cell differentiation^12,13^, and axon growth and guidance^14–17^. Microdevice-based approaches, which are used specifically for investigating axon guidance, have recently been reviewed comprehensively^18,19^.

Netrins are a small family of laminin-related, diffusible proteins that direct axon outgrowth during development^20^. Netrin-1, the most studied member of the family, was first shown to attract commissural neurons of the spinal cord to the ventral midline^21^; subsequently, the expression of netrin-1 has been observed in other structures of the central nervous system, such as the ganglionic eminence, the fimbria, the lateral septum, the external germinal layer of the cerebellum, and the retina^22^. In the hippocampus, in particular, netrin-1 deficiency leads to aberrant projections of the hippocampal commissural axons, as well as alterations in the ipsilateral entorhino-hippocampal connections and CA3-to-CA1 associations^23^. Netrin-1 is a bi-functional molecule: it attracts axons of some classes of neurons, while it repels several others^24^. Two classes of receptors, DCC and UNC-5, primarily mediate the attractive and repulsive responses of the axons^25,26^.

Most of the findings regarding the role of netrin-1 in axon guidance have come from knockout studies in mice and *in vitro* micropipette-based turning assays, mostly in *Xenopus*. However, pipette-based gradients do not enable precise quantitation. For example, in micropipette experiments, the absolute concentrations that the growth cone is exposed to have been estimated to be between 5 (Ref. 27) and 500 ng mL^−1^ (Ref. 4) (when the concentration in the pipette placed 100 μm away from the growth cone is 5 μg mL^−1^). Importantly, unless two pipettes are used, the concentration evolves over time because the use of a single pipette prevents the existence of a “sink” (a defined location near the cells where the concentration is known to be zero).

The complexities involved in the response of an axon to the concentration gradient of a cue, and its dependencies on other modulatory factors, require a platform that enables the (a) generation of precise and stable gradients, (b) tracking of individual growth cones over long periods of time, and (c) gathering of data from a large number (*n*>100) of axons in parallel to generate rich statistics. Microfluidic devices that generate flow-based gradients but are designed to shield neurons from flow-induced shear stresses can deliver stable, precisely controlled and dynamic gradients to the neurons at any point in time during the culture, and they are also compatible with automated live-cell tracking of the migrating axon-tip using phase contrast and epifluorescence microscopy^5^. In one such design (capable of analyzing *n*=10–25 axons per chip), *Xenopus* embryonic spinal neurons were challenged with a linear diffusible gradient of brain-derived neurotrophic factor (BDNF), and their exposure to fluidic shear was minimized by depositing them in micro-wells etched in the glass substrate^28^. Microfluidic methods and optical patterning have been used to deposit planar gradients of surface cues, such as laminin^15,29^, netrin-1, and BDNF^16^, which guided and modulated a directed extension of axons. However, when the substrates are pre-patterned with a gradient of the biomolecule cue, the cultured neurons are exposed to the gradient immediately after they are plated onto the substrate. Microfluidics has also been used to create concentration gradients of diffusible cues in hydrogels that also serve as scaffolds for neuron growth^17^.

We have previously described a microfluidic device based on “microjets”, which can generate gradients in an open reservoir with negligible flow, thereby subjecting the cells to very low shear stresses^30^. The device ensures high cell viability (at least 3 days) and gradient stability (a slope variation of <8% over 12 h)^31^. Importantly, the gradient formation is not sensitive to the molecular weight (M.W.) of the molecule used to create the gradient because the molecule of interest is mostly delivered via convective flow, therefore avoiding M.W.-dependent diffusive transport; thus, small-M.W. fluorescent tracers can be used to track the shape of the gradients of larger molecules because they follow very similar flow lines^31^. We showed that a majority of the cortical neurons extended axons towards the higher concentration of netrin-1 (Ref. 31). However, we also observed that when there were multiple neurons in a single gradient chamber, even in the presence of a netrin-1 gradient, many axons presumably grew towards adjacent cell bodies. This observation shows that the direction of axon growth can be influenced by cell-secreted factors.

We have designed a large-scale 16×64 (1024-element) array of microfluidic gradient chambers. We seed cells at an average cell density of 1 cell per chamber, which results in ~37% of the chambers being occupied by single cells, according to Poisson statistics. Notably, this seeding scheme decouples the possible effect of secreted factors from neighboring cells, as we reject the chambers occupied by multiple cells from the analysis. The neurons that attach outside the chamber (on top of the device) do not affect the in-chamber neurons because of two factors—distance and convection. According to the Stokes-Einstein relation (*L*^2^≈*Dt*), it would take ~18 s for a small secreted molecule (glutamate, M.W.=147, *D*=750 μm^2^ s^−1^) to travel 500 μm, which is the average distance between in-chamber and off-chamber cells. In ~18 s, the fluid volume of the bottom ~20 μm of the chamber (200×300 μm) will be displaced by fresh media (~1 nL) flowing in through the microjets (~200 nL h^−1^). Therefore, the gradient that is established by a continuous flow of fresh media effectively insulates the neurons in the chambers from factors secreted by off-chamber cells. Using live-cell time-lapse microscopy, we track the chambers that contain a single neuron and gather data from approximately 150–250 neurons per device; hence, in a single experiment, the device produces as much data as previous reports that gathered data from multiple (at least 3) experiments on microfluidic chambers containing many dissociated neurons^17,28,31^. This parallel, cell-friendly platform concurrently subjects a large number of hippocampal neurons to identical gradient conditions, enabling the researcher to obtain valuable insights into the dynamics and mechanisms of axon guidance. The device revealed a turning behavior that was dependent on the netrin concentration in a biphasic manner and the angle of orientation of the cell with respect to the netrin-1 gradient.

## Materials and methods

### Device fabrication

The microjet gradient generator array is made of poly-(dimethyl siloxane) (PDMS) (Sylgard 184, Dow Corning, Midland, MI, USA), a transparent biocompatible elastomer, which is molded off a master template fabricated on a silicon wafer using standard photolithography. The mold is made by sequentially exposing 2.5, 40, and 220 μm spin-coated layers of the photosensitive epoxy, SU-8 (Microchem, Newton, MA, USA), through high-resolution (20 000 dpi) masks, which are laser-printed on transparency films (CAD-Art Services, Bandon, OR, USA), using a mask-aligner (Quintel Q4000). Degassed PDMS (mixed at a ratio of 10:1 with the curing agent) is then poured onto the mold, excluded from the top of the tallest features by compressing it against a sheet of fluoro-polymer backed Mylar (Scotchpak Release Liner 9744, 3M, St. Paul, MN, USA), and then cured for at least 1 h in a convection oven at 70 °C. The cured PDMS membrane (250 μm) with the molded microstructures is peeled off from the silicon master, treated with oxygen plasma (660 mTorr, 60 W, and 60 s) in a plasma-cleaner (Diener Femto, Thierry Corp, Royal Oak, MI, USA), placed on a clean 48×65 mm No. 1 glass coverslip (Gold Seal, Thermo-Fisher Scientific, Waltham, MA, USA), and baked for 5 min on a hot plate at 75 °C. After the PDMS bonds to the glass surface, the Mylar sheet can be easily peeled off, leaving an array of open chambers. Blocks of PDMS (~5 mm thick and 5 mm in diameter) with 0.75-diam. holes, which are made using a biopsy punch (Harris Uni-Core, Ted Pella, Redding, CA, USA), are then bonded on top of the device to serve as fluidic inlet ports. The devices are then glued with double-sided acrylic-silicone adhesive tape (Adhesive Applications, Easthampton, MA, USA) on top of 40×50 mm openings, which are cut out in 100 mm polystyrene dishes (BD Biosciences, Franklin Lakes, NJ, USA) with a CO_2_ laser-cutter (M360, Universal Laser Systems, Scottsdale, AZ, USA). PDMS is applied on the edges and cured to ensure that the assembly is leak-proof.

### Modeling and simulation

We used finite-element modeling (COMSOL Multi-physics 3.3, COMSOL Inc., Burlington, MA, USA) to simulate the mass and momentum transport in our devices. We solved the Navier-Stokes equation for incompressible fluids and coupled that with the equations governing convection-diffusion processes for different initial conditions and geometric parameters of the device.

### Flow control and gradient application

To visualize the basic operation of the device, solutions of food-coloring dyes (Allura Red and FD&C Blue; 20 mM) were introduced into opposite ends of the microfluidic gradient generator array by applying a uniform pressure of 1–2 psi. For imaging the surface gradient, fluorescein (Sigma-Aldrich) (1 mM) mixed with Orange-G (Sigma-Aldrich, St Louis, MO, USA) (45 mM) in Tris-HCl (pH 8.0) was introduced at one end, and Orange-G (45 mM) alone was introduced at the other end of the array device using a syringe pump (Fusion 200, Chemyx Inc, Stafford, TX, USA) at different flow rates ranging from 50 to 300 μL h^−1^. The entire device was kept immersed in Orange-G (45 mM). For gradient experiments with neurons, netrin-1 (R&D Systems, Minneapolis, MN, USA) or c-myc-tagged netrin-1 (a gift from Tim Kennedy, McGill University, Montreal, QC, Canada) (100 ng mL^−1^) was introduced at one end, whereas Neurobasal media was flowed through the other end—both at 100 μL h^−1^ with the syringe pump. Trace amounts of Texas-Red and Cy5-conjugated bovine serum albumin (BSA) (Invitrogen, Carlsbad, CA, USA) (3 μg mL^−1^) were added on either side to visualize the onset of the gradient in the chambers.

### Device preparation for neuron culture

The completed device is treated with oxygen plasma (660 mTorr, 60 W, and 90 s) to make the surface hydrophilic and then sterilized with UV (in a Trans-Illuminator UV Box) for 15 min. The devices can be stored in sterile, de-ionized (DI) water till further use. Prior to seeding cells in the device, the device surfaces are treated with 50 μg mL^−1^ poly-D-lysine (Sigma-Aldrich Cat# P6407, St Louis, MO, USA) for 2 h at room temperature, followed by 10 μg mL^−1^ laminin-1 (Sigma-Aldrich, Cat# L2020) for 6 h at 37 °C. After thoroughly washing off poly-lysine and laminin-1, sterile filtered 0.1% bovine serum albumin (BSA) is flowed in the device for 1 h to block non-specific protein adsorption on the inside surfaces of the PDMS microchannels. Neurobasal media (Invitrogen) is flowed through the channels for 30 min to wash off the BSA solution. The entire device is then stored overnight (till the cells are ready for plating) in the incubator (5% CO_2_, 37 °C) and fully immersed in 10 mL of Neurobasal media. The CO_2_-rich environment in the incubator helps in dissolving any residual bubbles in the channels and microjets.

### Neuron collecting and culture

Hippocampal tissue is micro-surgically dissected from embryonic day 18 (E18) mice and (in some cases) stored in Hibernate media (Brainbits LLC, Springfield, IL, USA) supplemented with B-27 (Invitrogen) for up to 3 days after dissection. Primary neurons are collected from the hippocampi using a papain digestion kit (Worthington Biochemical, Lakewood, NJ, USA), following well-established protocols^32^. The dissociated neurons are suspended in Neurobasal media (Invitrogen), supplemented with 1X B-27 (Invitrogen), 0.5 mM GlutaMax (Invitrogen) and 100 U mL^−1^ penicillin-streptomycin (Invitrogen), and then passed through a 40 μm cell strainer. Once the cells are ready to be plated, most of the media is aspirated out from the dish with the device, leaving only a thin film of fluid to ensure fluidic continuity and thereby eliminate the chance of introducing any bubbles. The neurons are diluted to 12 000 cells per mL, and 10 mL of the cell solution is added on top of the devices to give a density of ~1 cell per 0.06 mm^2^ of surface area (or 16.67 cells per mm^2^). The cells were allowed to attach onto the surface for 24 h in the incubator (5% CO_2_, 37 °C) before gradients of netrin-1 are introduced.

### Device and gradient imaging

The testing and characterization of the microjet array devices with food-coloring dyes was performed using a stereomicroscope (Nikon). To quantify the gradient at the surface of the microjet array devices, we adapted a previously described imaging protocol using epifluorescence^33^. Orange-G, a non-fluorescent dye that absorbs energy at 488 nm (but not at 540 nm), competes with fluorescein in solution (1 mM) for the excitation energy. Therefore, at 45 mM Orange-G (with a 0.6 NA objective), the penetration length (distance from the surface at which the excitation intensity is 1/e times the incident intensity) calculated from the Beer–Lambert law is ~4.9 μm. Since the excitation decays exponentially from the surface, 95% (1–e^−3^) of the fluorescence intensity that is detected by the camera comes from a volume that is within ~15 μm of the surface.

### Time-lapse microscopy

For long-term imaging of the neurons under a gradient, the device was placed in an environmental chamber (Pathology Devices, Baltimore, MD, USA), which was fitted to the stage of an inverted epifluorescence microscope (Nikon TE 3000). The heat input was adjusted using a controller to maintain the temperature of the cell culture surface and the media at 37 °C during the course of the experiment. Pre-mixed 5% CO_2_ gas was bubbled through water and fed into the chamber. Holes were drilled on the side of the chamber to fit the tubing and connect it to the fluidic ports on the device. The microscope was fitted with Nikon’s Perfect Focus objectives that eliminate focus drift for long-term imaging. Phase contrast and fluorescence images were obtained with a 12-bit cooled CCD camera (ORCA ER, Hamamatsu, Japan). The stage-movement, the automated acquisition of the phase contrast and the fluorescence images for different chamber positions were controlled using the Nikon Elements software.

### Image analysis and statistical analysis

We developed a software package scripted in MATLAB (Mathworks, Cambridge, MA, USA) to perform semi-automated axon tracking, gradient quantification, data analysis, statistical testing and graph plotting. Image background corrections and contrast enhancements were performed using the FIJI (Image J, NIH, Bethesda, MD, USA) software. We used MATLAB and Microsoft Excel for our data and statistical analyses. Since the normality assumption for the data could not be met, we used parametric tests (performed on ranked data), such as the Mann-Whitney and Kruskal-Wallis tests, for testing all our null-hypotheses.

### Turning angle measurement

The turning angle measurement is schematically illustrated in Supplementary Figure S4b. At every time point *t*, we measured the angle *θ_t_* subtended by the growth cone with respect to the gradient direction. The turning angle is defined as the difference Δ*θ* between the angles that the growth cone makes with the gradient direction for successive time points (Δ*θ*=*θ_t_*−*θ*_*t*−1_), as schematically illustrated in Supplementary Figure S4b. A summation of the turning angles over the entire experimental time period resulted in the total angular change for a growth cone.

## Results

### Large-scale 1024 microjet array: Design and principle of operation

**Figure 2:**
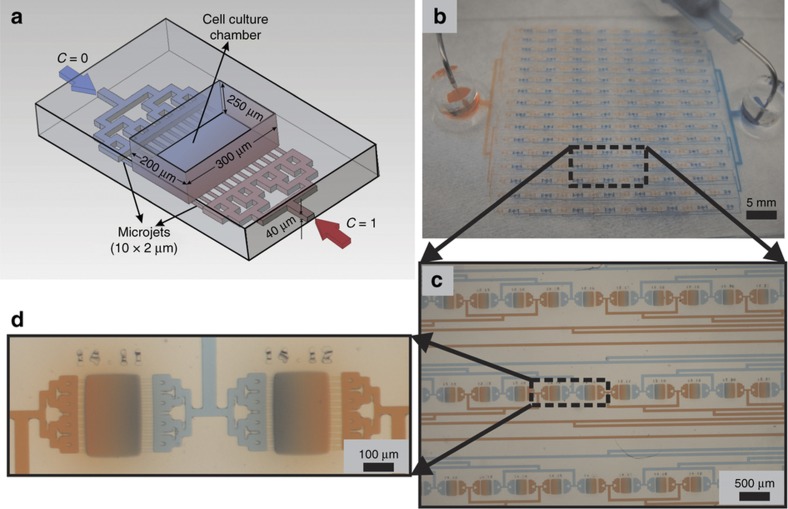
Microjet gradient array. (**a**) Schematic of one chamber of the microjet gradient generator array device. The entire device is made of 1024 such chambers. (**b**) A photograph of the device bonded onto glass and connected to fluidic lines (50 μm wide and 40 μm high) carrying 20 mM red and blue food-coloring dyes. (**c**) A micrograph showing 3 rows and 10 columns of the array, with chambers filled with opposing gradients of red and blue food-coloring dyes. (**d**) A magnified inset picture showing two chambers with a gradient.

Based on a previous design^31^, we developed a large-scale array of 1024 gradient generators that can be loaded with cells from the top using a pipette. The gradient generators are capable of creating identical concentration gradients in parallel with flow rates that are extremely low (~100 pL min^−1^ per chamber) and therefore benign to the cells. Each unit of the microjet array device (as observed in the schematic in Figure 2a), henceforth called the gradient chamber, is an open reservoir that is 250 μm high, 200 μm wide and 300 μm long. The gradient chamber is supplied on each side by a row of 12 microjets that are 2.5 μm high, 10 μm wide and 15 μm apart (Figure 2a). Very low volumes of fluid ejected from the microjets upon the application of a pressure head (~1 psi) or upon being driven by a syringe pump at a controlled flow rate (100 μL h^−1^) create a gradient in the open chamber, where on average, a single neuron is cultured. Each row of microjets from all the gradient chambers is connected to a common fluidic inlet port through 40 μm-high and 50 μm-wide microchannels that are routed through a binary distribution network to ensure equal hydrodynamic resistance from the port to each chamber. The gradient chambers are 1.2 mm apart, resulting in a 16-by-64 rectangular array. The device, which is plasma-bonded to a glass coverslip, is filled through needles connected to syringes driven by a syringe pump (Figure 2b).

### Gradient uniformity and stability

Gradient formation in the chambers can be visualized qualitatively using food-coloring dyes (Supplementary Figure S1a; the micrograph in Figure 2b shows a part of the device, and the magnified inset shows two of the chambers). Although the food-coloring dyes enabled us to quickly evaluate the chambers, notably, the low-magnification images captured by the stereomicroscope integrate different optical planes above the surface, thereby giving us only an indication of the formation of the gradient; these food-coloring dye images do not provide quantitative information on the surface gradient.

**Figure 3:**
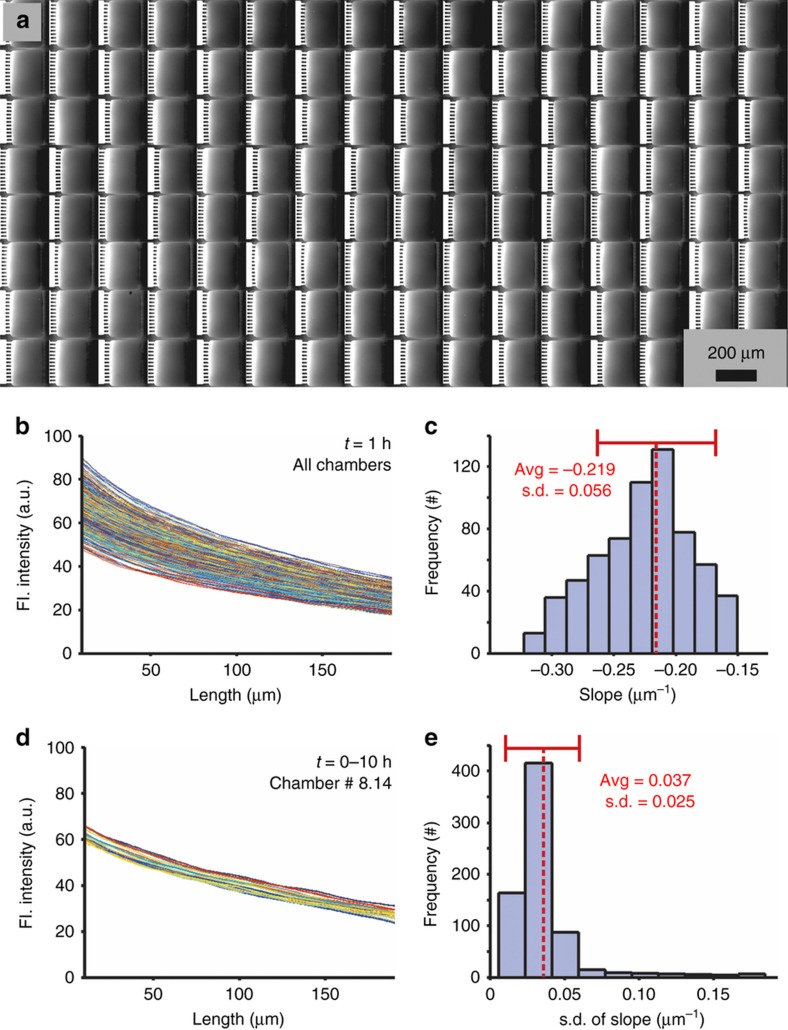
Surface gradient characterization. (**a**) A montage of cropped fluorescent images from 8×16 contiguous chambers of the microjet array device (1/8th of the total area of the device), where a gradient is formed by opposing streams of 1 mM fluorescein and 45 mM Orange-G. The fluorescence intensity in each image is proportional to the concentration of fluorescein just above the surface^33^. For clarity, alternate images have been flipped to ensure that the source of fluorescein is always lined up on the left. (**b**) A plot of fluorescent intensity line-traces (averaged over 10 pixels and obtained through the middle of the chamber) from all the working chambers in the device (*n*=720) taken 1 h after the initiation of flow. (**c**) A histogram plot of the best linear-fit slope angles of the line-traces in (**b**). The red dotted line denotes the average slope (−0.219 μm^−1^) with a standard deviation of 0.056 μm^−1^. (**d**) A plot of the fluorescent intensity line-traces from a typical chamber taken every 30 min for 10 h. (**e**) A histogram plot of the standard deviations of the slope angles over time for all the 720 chambers plotted in (**b**). The red dotted line denotes the average standard deviation of the slope (0.037±0.025 μm^−1^).

We quantitatively evaluated the uniformity, reproducibility and stability of the gradients generated along the surface of our device. The surface gradient is more representative of what the axons actually sense than the gradient in the bulk volume of the chamber above the neurons. Using a previously described protocol to image the surface fluorescence^33^, we characterized the surface gradients of all chambers in the device (Figure 3a shows an 8-by-16 region, Supplementary Figure S1b shows a larger 16-by-32 region from the same device). Line-scan traces (10 pixel wide) of the fluorescence intensity through the middle of each microjet (12 microjets per chamber) were averaged for every functional chamber in a particular device and plotted to demonstrate the uniformity of the surface gradient generated in the chambers (Figure 3b). A histogram plot illustrates the spread of the surface gradient slope angles across all the functional chambers (Figure 3c). The average slope was calculated as −0.219±0.056 μm^−1^ (indicated by a red dashed line in Figure 3c). It is important to note that even though the dye molecule accumulates in the bath over time, the concentration profile that the cells sense along the surface remains constant because of the continuous supply of fresh buffer and dye on either side of the chamber. The stability of the generated gradients over time was also determined by taking line-scan measurements of the surface gradient fluorescence intensity every 30 min for 10 h (Figure 3d shows the overlaid traces obtained at different time points along a line through the middle of a typical chamber in the array). To understand how the gradient fluctuated over time in all the functional chambers, we plotted a histogram of the standard deviation of the slope angles over time across all the chambers (Figure 3e). The narrow distribution of the standard deviation values (with an average±*σ*=0.037±0.025 μm^−1^, denoted by a red dashed line in Figure 3e) across all the chambers demonstrates the long-term stability of the gradient. This characterization is performed for each device as a calibration procedure to evaluate the gradient in each chamber both before and at the end of each experiment; since the pre-experiment and post-experiment gradient stability measurements do not differ by more than 5%, we conclude that we do not need to obtain gradient data while the cells are in the device. During the experiments, the fluid entering one side of the chamber was spiked with 5 μg mL^−1^ Texas-Red-conjugated BSA for real-time visualization of the onset of gradient formation (Supplementary Figure S1c). Since the epifluorescence microscope captures the signal from the entire volume of the chamber, and BSA gets deposited onto the surface over time, leading to an increase in the background signal, the tracer intensity plots do not accurately represent the surface gradient of the molecule. However, a constant slope (Supplementary Figure S1c) indicates the persistence of the surface gradient throughout the duration of the experiment. While the concentration gradients are not exactly identical across the chambers, the 2D gradient map of each chamber (obtained from the fluorescent images used for calibration) remains within small (<5%) error margins and is used to determine the concentrations that a particular axon is exposed to during the experiment. A small proportion (~2–7%) of the microjets supplying the chambers can get clogged over time, which results in the distortion or disappearance of the gradient in those chambers; in those few cases, the cells from these chambers are rejected from the analysis.

### Finite-element simulations of flow and concentration profiles

**Figure 4:**
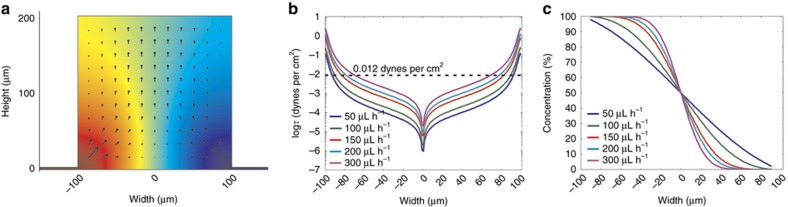
Finite-element modeling (FEM) of the device. (**a**) A two-dimensional FEM simulation (in COMSOL) of a vertical cross-section passing through a single microjet and the chamber, showing the concentration profile of netrin-1 (color map) and the flow velocity in the chamber (black arrows, with the arrow-heads indicating the flow direction and with the length proportional to the magnitude of the flow velocity). In this particular simulation, the flow rate through a single microjet was 2.30 pL s^−1^ (which corresponded to a flow rate of 100 μL h^−1^ through the entire device). (**b**) A plot of the fluidic shear stress exerted on the surface of the chambers for different flow rates through the device. The dotted line denotes the shear stress limit (0.012 dynes per cm^2^) that elicits turning in mammalian growth cones. (**c**) A plot of the steady-state concentration profiles of netrin-1, 10 μm above the surface of the chamber, for different flow rates.

We created a finite-element model of the device to determine the optimal flow rates needed to produce a linear gradient on the surface without exerting cell-harming shear stresses. A 2-dimensional geometry, drawn by taking the vertical cross-section of a pair of opposing microjets and the central gradient chamber, was used to simulate the flow characteristics and concentration profiles in one unit of our device. The color map in Figure 4a describes the steady-state concentration of the diffusing species in the chamber, whereas the magnitude and direction of the arrows represent the velocity field. The inlet fluid velocity boundary conditions (for the solution in Figure 4a) were made symmetric on either side, and they corresponded to a total flow rate of 100 μL h^−1^ into the device (2.30 pL s^−1^ per microjet or 92 μm s^−1^ per microjet). After coming out of the microjets, the flow lines are primarily directed upwards in the open gradient chamber (Figure 4a)—therefore, the horizontal component *X* of the fluid velocity (along the *XZ* surface) rapidly decreases towards the center of the gradient chamber (Supplementary Figure S2). Concomitantly, the shear stress on the cell culture surface decreases towards the center of the gradient chamber in our device. This observation can be understood by noting that the shear stress *τ* is proportional to the derivative taken along the surface normal *Y* of the *X* velocity component *u*_*X*_ parallel to the shear direction, *τ*=μ*(∂*u*_*X*_/∂*y*). A logarithmic plot of the shear stress (Figure 4b) at different flow rates show that for the middle 50% of the device, it is less than 10^−3^ dynes per cm^2^. Previous studies with optically actuated particles have shown that rotational shear stresses greater than 0.012 dynes per cm^2^ can elicit turning in mammalian growth cones^34^. At our operating flow rates, in 90% of the gradient chamber surface area, the shear stress is less than 0.012 dynes per cm^2^ (represented by the dotted line in Figure 4b). The simulation results also demonstrate that the steady-state gradient at the surface is sigmoidal in nature, with the central linear region spanning ~35–85% of the chamber, depending on the flow rates used (Figure 4c). Changing the flow rates alters the slope of the concentration profile, with higher flow rates producing a steeper gradient but a narrower linear region (Figure 4c). However, the simulations assume a continuous slit/microjet along the width of a chamber, whereas in reality, the microjets are 10 μm wide and separated by 15 μm. Therefore, the concentration slopes are over-estimated in the 2D simulations. The simulations show that while the device is capable of generating linear concentration gradients with different slopes, it does so by exposing the cells to extremely low shear stresses that do not compromise the long-term viability of the cells. We selected 100 μL h^−1^ as the optimal flow rate at which to operate our microjet array device, as it generates a linear gradient over 90% of the chamber area without subjecting the axons to detrimental shear stress levels.

### Hippocampal neuron culture in microjet array device

Then, we evaluated the ability of the microjet device to support the culture and differentiation of individual, dissociated primary neurons in culture. *In vitro* cultured primary neurons isolated from the hippocampus of an E18 mouse undergo distinct morphological changes during development, which have previously been characterized in detail^35^. In our device, neurons are grown in open reservoirs on laminin-coated glass substrates. Our substrate selection was based on evidence indicating the expression of laminin in a developing hippocampus^36^. The open reservoir design ensures that the equilibration of gases, pH and humidity occurs at similar rates as those in non-microfluidic formats, such as petri dishes or multi-well plates, which have been optimized for conventional cell culture. Indeed, the resultant hippocampal cultures in our microjet devices follow the typical development patterns of growth in standard dish cultures^31^. The time required for axon-dendritic differentiation ranged from between 12 and 30 h after plating (similar to earlier reports using tissue culture dishes^35^). Therefore, we started the gradient exposure experiments 24 h after plating, at which time most neurons had a neurite that had grown significantly longer than the others.

To achieve an average cell density of one cell per well, the neurons were diluted to obtain a surface density of 1 cell for every 200×300 μm area. The number of neurons in the chambers followed the Poisson distribution (Pr (*X*=*k*)=*λ*^*k*^*e*^−*λ*^/*k*!, where *X* is a discrete random variable, *k* is a non-negative integer and *λ* is the expected value of *X*). Therefore, the probability of having a chamber with exactly 1 cell (*λ*=1, *k*=1) is 36.7%, exactly 2 cells (*λ*=1, *k*=2) is 18.4%, and more than 2 cells (*λ*=1, *k*>2) is 8.2%. In accordance with the Poisson probability mass function, we observed that the percentage of chambers with single cells, 2 h after plating, was 36.13±3.22% (Supplementary Figure S3a). However, not all cells that are collected from the tissue survive the dissociation procedure (~80% viability), and ~67% of those cells have a distinct neurite, which is extended 24 h after plating (Supplementary Figure S3a). in addition, chambers with a non-linear gradient due to fabrication defects or clogging (~10%) or a cell with its neurite tip close to the edges (~10%) need to be excluded from the analysis, and this results in a further decrease in the number of “eligible” data points per experiment (Supplementary Figure S3b). The large-scale format and the open-top architecture of the device ensures that even when we simply add dissociated neurons using a conventional pipette, we can obtain ~160 chambers with single, isolated neurons with clearly discernible neurite outgrowth at 24 h (Supplementary Figure S3b).

### Netrin-1 acts as a growth factor for hippocampal neurons

**Figure 5:**
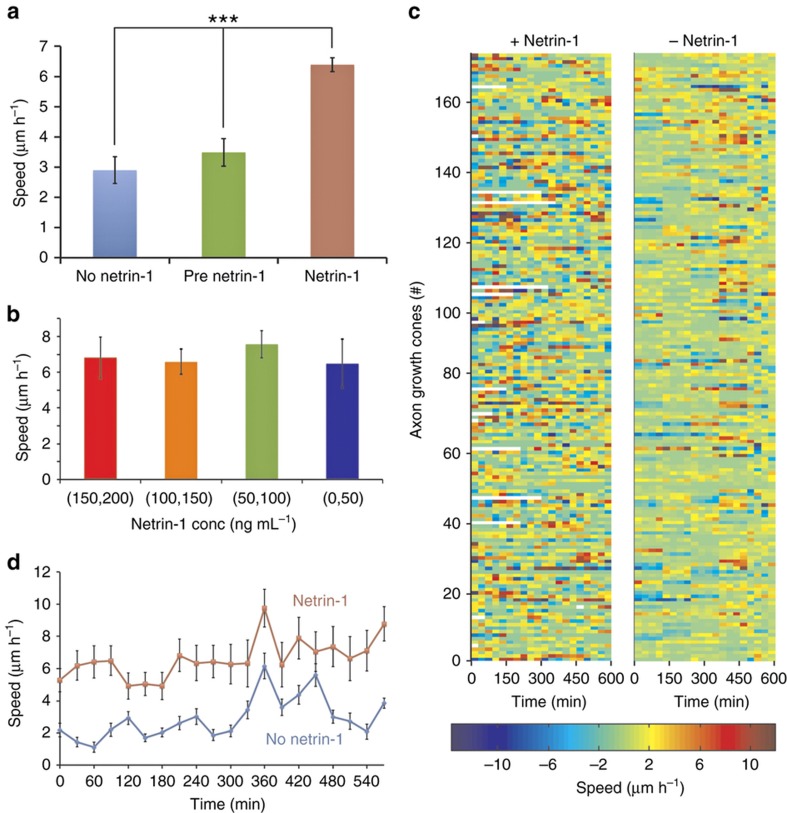
Migration speed of the axons. (**a**) Bar chart showing the average migration speed of the axons (*n*=406 neurons from a total of 3 experiments) in a netrin-1 gradient (maroon) compared to their speed before the application of netrin-1 (green) and in separate control experiments (*n*=212) without netrin-1 (blue). Error bars denote s.e.m. The difference in the average speed of the axons in the presence of the netrin-1 gradient compared to the speeds in the pre-netrin-1 and no netrin-1 controls was statistically significant (*P*-value<0.01, using the Mann–Whitney test). (**b**) Bar chart showing the average migration speeds of the axons exposed to different concentrations of netrin-1 because of their positions in the chamber. Error bars denote s.e.m. (**c**) A color heat-map of the migration speeds of individual neurons (*y* axis) over the entire duration of an experiment (*x* axis)—the left-panel shows neurons exposed to a gradient of netrin-1 and the right panel shows neurons in no netrin-1 controls. (**d**) A plot of the average migration speeds over time of neurons in a netrin-1 gradient (red line) and in no netrin-1 controls (blue line). Error bars denote s.e.m. for *n*=406 neurons from three experiments.

In retinal ganglion axons, netrin-1 has been reported to cause a dose-dependent increase in the neurite growth rate^37^. Therefore, we examined whether netrin may similarly act as a growth factor for hippocampal axons. We conducted three separate experiments in our microjet array device, where we exposed a total of 593 dissociated single hippocampal neurons (collected from different animals on 3 different days and pooled together) to a concentration gradient of netrin-1 (1 ng mL^−1^ μm^−1^) for up to 10 h under the same target conditions. Before applying a gradient of netrin-1, we flowed in culture media through both ports of the device at 100 μL h^−1^ for 2 h; the images captured during this timeframe constituted our pre-netrin-1 axon growth control data. Of the 593 neurons imaged, 406 (68%) neurons were situated in “eligible” chambers, and they extended axons by at least 3.3 μm (Supplementary Figure S3b). We compared the growth cone migration speed of these 406 neurons after the application of netrin-1 with their speeds prior to gradient application and also with the migration speeds of 212 neurons in a control set of experiments, where only media (without netrin-1) was flowed through the microjets. For every measured time point, we only considered the growth cone displacements larger than 3.3 μm in length as “displacements”. Of these displacements, the ones that occurred within an angular spread of 240° with respect to the growth cone direction were defined as “extensions”, while the others were defined as “retractions” (schematically illustrated in Supplementary Figure S4a). The mean migration speed in the presence of netrin-1 was determined as 6.39±0.35 μm h^−1^, whereas the speeds prior to netrin-1 application and in the no netrin-1 controls were 3.49±0.46 and 2.90±0.44 μm h^−1^, respectively (Figure 5a). The 1.8-fold increase in the growth rate upon the application of netrin-1 was statistically significant (*P*-value<0.01, Mann–Whitney test). We did not observe any significant difference in the migration speeds of the growth cones that were exposed to different absolute concentrations of netrin-1 due to their position in the gradient chamber (Figure 5b). We used a color map (Figure 5c) to visualize the netrin-1-induced variation in the speeds of individual axons (along the *y* axis) over time (along the *x* axis) and compared it with the control neurons that were not exposed to netrin-1. A cursory look at the data shows that in the presence of netrin-1, the axon growth cone migrates in bursts and shows periods of high-speed extensions followed by slow growth or even retractions. This behavior can probably be attributed to a synergistic effect of the internal state of the cell and the transduction of signaling cascades induced by netrin-1. By contrast, the no netrin-1 controls show a more uniform low-speed and less dynamic migration. From the plot of the average migration speeds for each time point (Figure 5d), it is evident that the speed increases with time both in the presence and absence of a netrin-1 gradient. For instance, the migration speeds were 5.88±0.75 and 2.11±0.62 μm h^−1^ for the axons in the netrin-1 gradient and the control, respectively, in the first 5 h (0–300 min), whereas they were 7.34±1.17 μm h^−1^ and 3.69±1.37 μm h^−1^, respectively, in the later 5-hour period (300–600 min). However, for all time points, the migration speed in the presence of netrin-1 was 2.62±1.05 times the speed in the absence of netrin-1. Although we did not see any evidence of a dose-dependent response (at ~1 nM or above), we observed a netrin-1-induced, statistically significant increase in the migration speed of the axons, which suggests that the switch to a higher migration speed possibly occurs with more than nanomolar sensitivity.

### Turning of hippocampal axons in netrin-1 gradient

**Figure 6:**
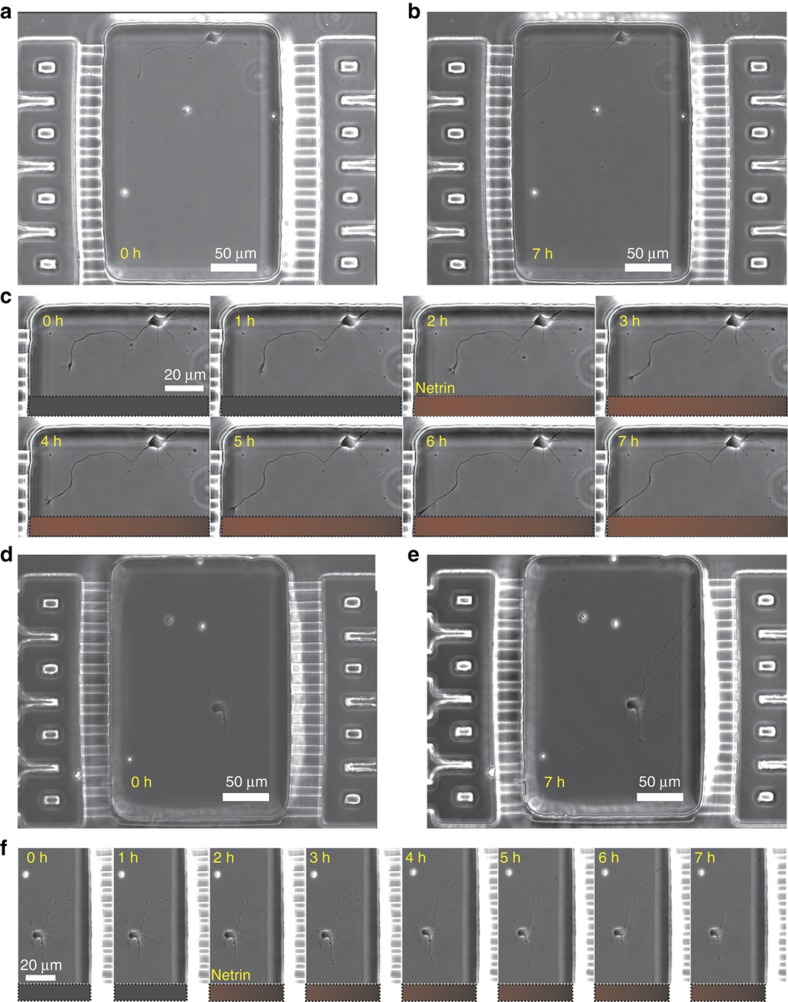
Hippocampal neuron culture in gradient chambers. (**a** and **b**) A phase contrast micrograph of a typical chamber with a single neuron turning towards the gradient 2 h before and 5 h after the application of a gradient of netrin-1 (1 ng mL^−1^ μm^−1^). (**c**) A montage of magnified phase contrast micrographs of the neuron in the chamber shown in (**a**) at different time points during the application of the netrin-1 gradient. The fluorescent image from the BSA-Texas-Red signal over the same region of the chamber is shown below each time point image of the neuron. (**d** and **e**) A phase contrast micrograph of a typical chamber with a single neuron turning away from the gradient 2 h before and 5 h after the application of a gradient of netrin-1 (1 ng mL^−1^ μm^−1^). (**f**) A montage of magnified phase contrast micrographs of the neuron in the chamber shown in (**d**) at different time points during the application of the netrin-1 gradient. The fluorescent image from the BSA-Texas-Red signal over the same region of the chamber is shown below each time point image of the neuron.

Then, we asked what the effect of a netrin-1 gradient would be on the hippocampal axons grown in the microjet device. Since netrin-1 is a secreted molecule, earlier studies assumed that netrin-1 signals in its soluble form, and so it was delivered in its soluble form. However, more recently, Moore *et al*. have shown that growth cones respond to netrin-1 only when it is bound to the substrate^38^. Our study does not delve into the nature of the netrin-1-substrate binding; here, the microjets are used as an *in vitro* mimic of the cell source that secretes netrin-1 *in vivo*, while the glass substrate acts as the intermediate signal binding substrate. In all of our experiments, the gradients were simply started without netrin and switched to netrin gradients at a particular point in time. The automated live-cell, time-lapse microscopy captured images of the neurons every 20 or 30 min (Supplementary Movies). Figures 6a, b, d and e show example micrographs of a chamber with a neuron turning towards or away from the gradient at 0 and 7 h. The time-lapse images (Figures 6c and f) show examples of neurons growing towards or away from the direction of the gradient, respectively. The trajectories of 406 (68%) neurons, which were situated in “eligible” chambers and extended axons by at least 3.3 μm (10 pixels), were plotted together in a graph, with the origin coinciding with the axon-tip coordinates at the beginning of the gradient application (Supplementary Figure S5). These trajectories, as well as the end-points of the axon tips, show no significant difference compared to a randomly generated set of points from a uniform distribution. We found that 48.5% (197) of the axons that migrated by at least 10 μm (approx. one cell body) were extended towards the gradient (Supplementary Figure S6a). Furthermore, in 51.5% of them (210), the first turn was towards the gradient (Supplementary Figure S6b). Interestingly, in 28.5% of the axons that extended by over 50 μm (70 out of 246), the first turn was not representative of the final turning behavior of the axons (Supplementary Figure S6c), which indicates that inferences drawn from the initial response of the axons in a gradient may not always be representative of their long-term behavior.

### Growth cone turning depends on absolute concentration of netrin-1

**Figure 7:**
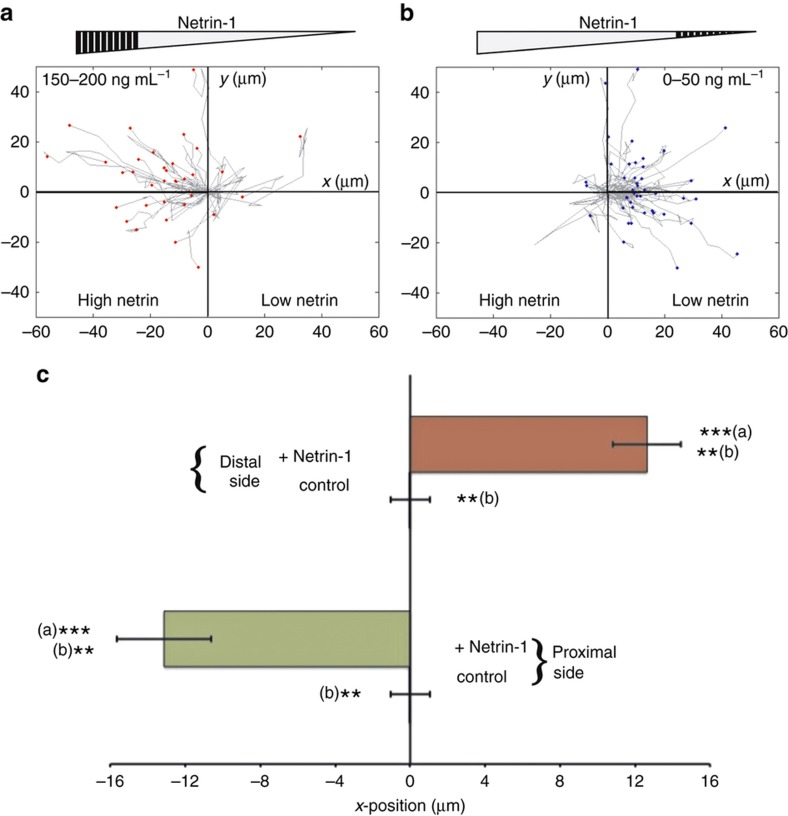
Biphasic behavior of growth cone trajectories. (**a** and **b**) A plot of the trajectories of all the axons that were exposed to high and low concentration ranges of netrin-1 (denoted by the shaded region of the gray triangle on the top). The origin in these plots denotes the initial position of the growth cones before the beginning of the gradient application. (**c**) A plot of the average displacement, along the axis of the microjets, of the growth cones in the experimental and control devices, from the proximal (150–200 ng mL^−1^) and distal (0–50 ng mL^−1^) sections of the chamber (*n*=40 and 33 in distal and proximal experimental chambers, respectively, and 47 in control chambers, where the two sections are equivalent and therefore combined). Error bars denote s.e.m. ***(a) indicates a *P*-value<0.005 between the proximal and distal section axons exposed to the netrin-1 gradient. **(b) indicates a *P*-value<0.01 between the axons of the experimental and control devices. The Mann–Whitney test was used to determine the statistical significance.

While the analysis in the previous section indicated that a linear gradient of netrin-1 did not appear to direct the extension of the hippocampal axons in a particular direction (Supplementary Figure S5), we investigated whether the absolute concentration observed by the growth cone could have played a role in determining the response to the netrin-1 gradient. We sub-divided the axons that extended by over 10 μm (*n*=326) into four groups, according to the absolute concentration of netrin-1 that the growth cone was exposed to at the initial time point. Strikingly, we found that the axons with growth cones initially exposed to the highest concentrations of netrin-1 (150–200 ng mL^−1^) grew towards the netrin-1 source (Figure 7a), whereas the axons with growth cones exposed to low concentrations of netrin-1 (0–50 ng mL^−1^) grew away from the gradient (Figure 7b). To quantify this difference, we measured the average displacements of the axon growth cone along the direction of the gradient, which were −13.13 and 12.64 μm, respectively, for the high and low concentration populations (Figure 7c). These results again were statistically significant (*P*-value<0.005). To confirm that this difference in response was not an artifact of the experimental setup or flow conditions, we compared these results with the trajectories of growth cones positioned in the quarters of the chambers closest to the microjets in control experiments, where only cell culture media flowed through both sets of microjets. In the absence of a gradient, the chamber is symmetric about the centerline. Since we cannot distinguish between the quarters closest to the microjets on either side of the chamber, for our analysis, we combine the data from these two regions in the control chambers. Without netrin-1, there was no obvious directionality to the trajectories (Supplementary Figure S7), and the average displacement was −0.02 μm (Figure 7c), which was significantly different (*P*-value<0.01) from the corresponding trajectories in the netrin-1 experiments. Thus, hippocampal axons presumably are unequivocally attracted at higher concentration gradients and repelled at lower concentration gradients.

**Figure 8:**
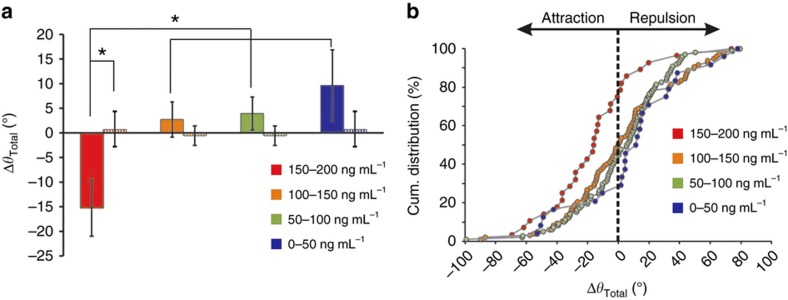
Dependence of the turning of growth cones on the absolute concentration of netrin-1. (**a**) Bar chart showing the average total turning angle of a growth cone exposed to different ranges of concentrations of netrin-1: (150–200) (red), [100–150] (orange), [50–100] (green), and (0–50) (blue), in ng mL^−1^; all neurons were exposed to a linear gradient of 1 ng mL^−1 ^μm^−1^ (solid bars) or no gradient (striped bars). Error bars denote s.e.m. (*n*=40, 127, 124, and 35 for the (150–200), (100–150), (50–100), and (0–50) ranges, respectively, from three experiments). The asterisk denotes the statistical significance (*P*-value<0.01, using the Kruskal–Wallis test). (**b**) A cumulative distribution plot of the turning angles of growth cones exposed to different absolute concentrations of netrin-1. The *y* axis represents the percentage of growth cones with turning angles less than or equal to a given angular value in the *x* axis. Negative values indicate a turn in the direction of the gradient. For example, of the growth cones exposed to the netrin-1 gradient at 150–200 ng mL^−1^ (red), 78.5% were attracted; whereas of those exposed to 0–50 ng mL^−1^ (blue), only 29.1% were attracted.

Next, we examined whether the concentration dependence that we observed in the trajectories of the axons was also reflected in the actual turning angle of the axons from the high and low concentration sides of the chamber. The method used to determine the turning angle is schematically illustrated in Supplementary Figure S4b and described in the MATERIALS AND METHODS section. We found that the mean turning angle for the axons exposed to the highest concentrations of netrin-1 (150–200 ng mL^−1^) was −15.25°, that is, they mostly turned towards the gradient (Figure 8a). For the other concentration ranges, the mean axon turning angles were 2.73° (100–150 ng mL^−1^), 3.93° (50–100 ng mL^−1^) and 9.62° (0–50 ng mL^−1^). In the no netrin-1 control experiments, the mean turning angles were 0.68±3.55° and −0.57±1.96° for the parts of the chamber next to the microjets and in the middle half of the chamber, respectively (Figure 8a). This concentration-dependent trend was consistent even when we looked at just the first turn of the growth cone after the application of netrin-1 (Supplementary Figure S8). In addition, the cumulative distribution of the turning angles (Figure 8b) showed that 78.5% of the axons whose growth cones were exposed to the highest concentrations of netrin-1 (150–200 ng mL^−1^) turned in the direction of the gradient compared to 50.5%, 44.8%, and 29.1% of the axons in the 100–150, 50–100, and 0–50 ng mL^−1^ concentration range groups, respectively. The difference between the turning response of the axons exposed to 150–200 ng mL^−1^ netrin-1 and that from the others and the no netrin-1 control group was statistically significant (*P*-value<0.01, using the Kruskal–Wallis test). The results above indicate a biphasic response to netrin-1: at concentrations from 150 to 200 ng mL^−1^, a linear gradient of 1 ng mL^−1^ μm^−1^ attracts the growth cones towards the source of netrin-1, while at concentrations less than 50 ng mL^−1^, the same gradient appears to be repulsive.

### Growth cone turning depends on angle at which gradient is incident

**Figure 9:**
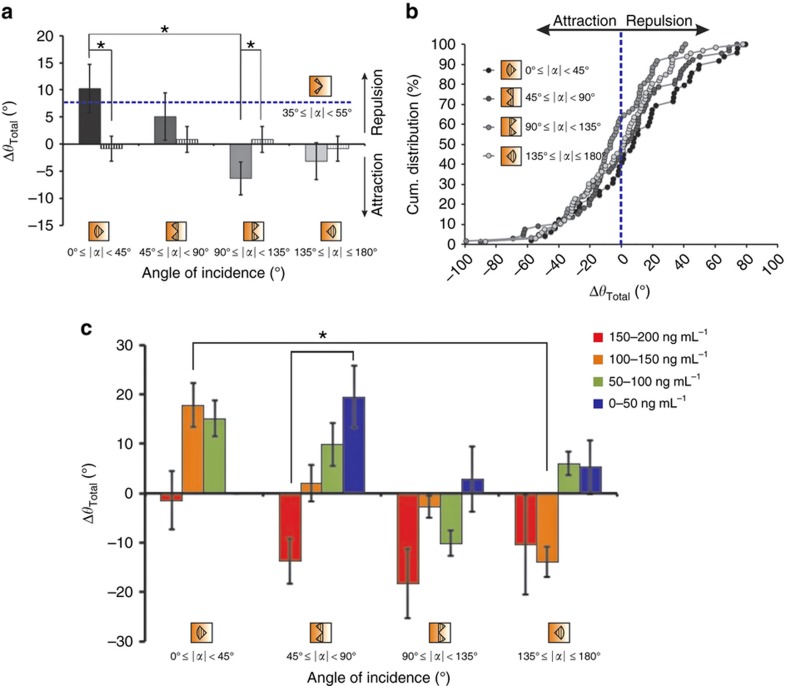
Relationship between the turning angle and the angle of incidence of the gradient. (**a**) Bar chart showing the mean total turning angle of a growth cone exposed to different angles of orientation (45° sectors) of the netrin-1 gradient (solid bars) compared to the no netrin-1 control group (striped bars). The mean turning angle in axons oriented 35°–55° to the netrin-1 gradient is indicated (blue dashed line) on the plot. The axons exposed to the gradient at an angle 0°–45° (black, *n*=71) were significantly different from those at an angle 90°–135° (light gray, *n*=83) (*P*-value<0.01, using the Kruskal–Wallis test). (**b**) Cumulative distribution plot of the total turn (angle) of growth cones exposed to different angles of orientation (45° sectors) of the netrin-1 gradient. (**c**) Bar chart showing the average total turning angle of a growth cone exposed to different angles of orientation (45° sectors) of the netrin-1 gradient and different concentration regions (0–50, 50–100, 100–150, 150–200 ng mL^−1^). Error bars denote s.e.m. (*n*=5–29). The asterisks denote the statistical significance (*P*-value<0.01, using the Kruskal–Wallis test). Negative values indicate a turn in the direction of the gradient.

In micropipette-based protocols, the pipette is always positioned at 45° with respect to the advancing growth cone^27^. Therefore, it is not possible to determine whether the angle of incidence of the gradient plays a role in the response of the growth cones to the gradient. In our microfluidic setup, the fixed direction of gradient generation and the random seeding of the neurons resulted in a uniform distribution of the angle of orientation of the axons with respect to the gradient (Figure 1). In addition, the large-scale data set enabled us to look into the relationship between the growth cone turning and the angle of incidence of the gradient. Similar to the earlier concentration analysis, we sub-divided the data set of growth cone angles (*α*) at the initial time point *t*_0_ into four sub-quadrants (consisting of 45° sectors) based on the angle of incidence of the gradient at *t*_0_, and we plotted the average turning angle over the entire experimental period of 10 h for neurons in each of these categories (Figure 9a). Surprisingly, there was a statistically significant dependence (Kruskal–Wallis test) on the initial angle of incidence of the gradient. On average, growth cones that were oriented away from the gradient (90–135°) were attracted (−7.13±3.35°, *n*=83), whereas the ones that were oriented towards the gradient (0–45°) were repelled (11.87±5.31°, *n*=71; Figure 9a). The cumulative distribution plot showed that 64.5% of the growth cones oriented at an angle of 90–135° turned in the direction of the gradient, whereas only 38.7% of those oriented at an angle less than 45° to the gradient were attracted (Figure 9b). For the other two populations (at 45–90° and 135–180°), the difference was not significant: 46.9% (*n*=85) and 47% (*n*=87) of the growth cones turned towards the gradient. In the absence of netrin-1, the angle of incidence was defined with respect to the flow vector. Since the flow rate and direction were the same at either end of the chambers, the (0–45°) and (135–180°) sectors were equivalent (mean turning angle=−0.85±2.32°), and so were the (45–90°), and (90–135°) sectors (mean turning angle=0.87±2.40°). In contrast, micropipette studies orient the gradient direction at approximately 45° to the advancing growth cones; therefore, we also looked at the turning angle of neurons with growth cones at angles between 35° and 55° (gray dashed line in Figure 9a) and found that they were repelled (8.69±4.62°, *n*=25), which contradicts earlier findings. Most studies of single growth cone behavior to netrin gradients have focused on a short time window after the application of netrin. Therefore, we asked whether the first turn of the growth cone, after the application of the netrin-1 gradient, would also show a dependence on the angle of incidence similar to the cumulative response—with growth cones oriented away from the gradient being attracted (on average) and the ones oriented towards being repelled (Supplementary Figure S9). In summary, on average, the first turn of the axon in response to a netrin gradient behaves similar to the cumulative trajectory; at larger angles, particularly between 90° and 135°, the axons were attracted, and at the smallest angles (less than 45°), the axons were repelled.

Then, we asked how this biphasic dependence of the turning behavior on the angle of incidence is related to the biphasic dependence on the absolute concentration that was described earlier. We further sub-divided the growth cone population along both category axes to determine if there were particular combinations of concentration and incident angle conditions that favored a particular turning behavior (Figure 9c). The growth cones in the higher netrin-1 concentration region (150–200 ng mL^−1^) strongly turned towards the gradient for all angles of incidence, except for angles less than 45° (44.5% of the growth cones in the 150–200 ng mL^−1^ region that turned away (Figure 8b) were oriented at an angle less than 45° to the gradient). Likewise, the growth cones oriented at an angle of 90–135° to the gradient were mostly attracted, irrespective of the concentration range they were in. The growth cones at the highest concentrations (150–200 ng mL^−1^) are most strongly attracted (−18.3±7°) at angles from 90 to 135°, while the growth cones at the lowest concentrations (0–50 ng mL^−1^) are most strongly repelled (19.5±6.2°) at angles from 45 to 90°. At the intermediate concentrations, both attraction and repulsion was observed, with attraction observed at higher angles and repulsion observed at lower angles. When the angle of incidence was between 45° and 90°, there was a linear dependence between the percentage of growth cones that were attracted (as well as the mean turning angles, as shown in Figure 9c) and the absolute concentrations of netrin-1 (in particular, 16.7, 40.7, 50.0, and 70.0% of the growth cones exposed to the netrin-1 gradient at 150–200, 100–150, 50–100, and 0–50 ng mL^−1^, respectively, were attracted). At netrin-1 concentrations of 100–150 ng mL^−1^, a similar linear trend (orange bars in Figure 9c) was observed between the mean turning angles of growth cones and their angles of orientation (17.85°, 2.01°, −2.7°, and −13.88° when the gradient was incident at angles 0–45°, 45–90°, 90–135°, and 135–180°, respectively).

In summary, using our gradient generator array we have shown that in mammalian hippocampal neurons, both the absolute concentration and the angle of incidence are important parameters in determining the turning response of a growth cone to a linear gradient of netrin-1. With respect to the concentration, growth cones closer to the source of netrin-1 (those exposed to a high concentration) are strongly attracted, and those far from the source (exposed to a low concentration) are repelled. With respect to the angle of incidence, growth cones oriented towards the gradient (less than 45° with respect to the direction of the netrin-1 source) are strongly repelled.

## Discussion

We report an integrated platform that can assay axonal growth over a large number of individual, isolated, dissociated mammalian primary neurons in parallel in the same chemotropic field. The number of assayed cells per experiment (~200) is around an order of magnitude more than that from previous reports with dissociated neurons^16,27,28,39^. The flow-induced shear stress in our device was low enough to sustain growth cone motility for 10 h and, in addition, not bias the growth cone turning; on occasion, we have observed the growth cones to even turn towards the direction of the flow. Since this platform was demonstrated to be very benign to primary mammalian neurons, which are a notoriously delicate cell type to maintain in culture, we anticipate that it should be easily adapted to *in vitro* migration studies using other cell types, such as cancer cells, stem cells, and immune cells. Traditional micropipette-based axon guidance studies have been limited to testing one condition at a time, and therefore, they have always relied on small data sets that usually reduce the statistical power of the tests^2,27^. Collagen gel assays, which analyze neurite outgrowth from explants, have shown the power of large data sets in unveiling interesting relationships between neurite guidance and the chemotropic field^40,41^ and challenging established mechanistic paradigms of axon growth and guidance^42^. Earlier microfluidic approaches^17,28^ have been able to study a higher number of dissociated neurons per assay (10–50 on average). As conceptually depicted in Figure 1, our device creates a quantifiable and stable linear gradient, but it is often difficult to directly compare the results that we observe in microfluidic systems such as ours with those obtained from micropipette assays.

One of our principal findings is how the absolute concentration of netrin-1 affects the turning response of hippocampal growth cones in a netrin-1 gradient field; high concentrations lead to attraction, and low concentrations lead to repulsion. A concentration-dependent biphasic turning behavior with netrin-1 has not been previously reported in hippocampal or other neurons; however, such a behavior has been observed in retinal ganglion cells in response to the morphogen Shh^43^. Our finding would not have been possible without the large-scale and parallel format of our device, which enabled us to gather time-lapse data from over 400 neurons in three experiments. This finding also would not have been apparent without the ability of our device to undergo long-term exposure to a stable netrin-1 gradient for over 10 h.

In other studies of mammalian neurons, attraction and repulsion were found to be due to the action of different netrin receptors. Here, we found evidence for the expression of both receptor types in all neurons. What might be the mechanism of this biphasic behavior? Interestingly, in studies with *Xenopus* neurons, a transition between attraction and repulsion could result from manipulations of second-messenger signaling^27,44,45^. The response to netrin-1 can also change from attraction to repulsion depending on the specific developmental stage—for example, retinal ganglion cells of *Xenopus*, which are attracted by netrin-1 early on, are repelled by it at a later developmental stage^46^. Furthermore, axonal growth cones of cultured *Xenopus* spinal neurons exhibit adaptation during chemotactic migration, and they undergo phases of desensitization and resensitization in the presence of increasing basal concentrations of netrin-1^47^. A possible mechanism for the biphasic response might lie in the different sensitivities of the two types of netrin receptors. The presence of both DCC and UNC-5 receptors has been demonstrated in hippocampal neurons both in our study and in earlier reports^48^. In *Xenopus* spinal neurons, the netrin-1-dependent hetero-dimerization between UNC-5 and DCC can lead to a long-range repulsive guidance^49^, while the ligand-gated homo-dimerization of DCC governs attraction^50^. Presumably, in our experiments both the activated heterodimer (UNC-5/DCC) and the homodimer (DCC alone) would be present. The reported dissociation constant of DCC-netrin (~3 nM)^26^ is lower than that of UNC5-netrin (~20 nM)^25^, but the DCC-UNC-5-mediated chemo-repulsion is unaffected even when the ligand-binding extra-cellular domain of UNC-5 was truncated^49^. Therefore, it is unclear what the functional EC_50_ might be. In our experiments, the concentration range (150–200 ng mL^−1^) at which we see pronounced attraction corresponds to a molar concentration (~3 nM) that is close to the dissociation constant of DCC-netrin (~5 nM)^26^, at which about half the DCC receptors should be in the bound state. In an earlier report with bulk hippocampal axons in a gel-based gradient, netrin-1-mediated attraction was not observed at concentrations<1 nM (which corresponds to the lower end of our concentration range, 0–50 ng mL^−1^), even when the neurons were transfected to overexpress DCC^17^. Thus, we would not expect strong netrin-mediated attraction at the lower netrin concentrations at which we predominantly observed repulsion. Moreover, the fact that UNC-5 works at a long range in *Drosophila* as a DCC heterodimer^51^ suggests that this mechanism of repulsion is probably sensitive to lower concentrations of netrin-1. Therefore, consistent with an earlier observation that the ratio of the DCC and UNC5 receptors determined the behavior of the hippocampal axons to netrin-1^48^, we hypothesize that repulsion due to heterodimers may act at the lower concentration ranges in our experiment, while attraction due to homodimers requires the higher concentration range.

The underlying mechanism for the biphasic response we observe could potentially arise from the action of protein kinase Cα (PKC-α)^52^, as implied in studies of netrin in *Xenopus*. Netrin-1 induces PIP_2_ hydrolysis in a DCC-dependent (and UNC-5-independent) manner, thus activating PKC-α through the formation of diacylglycerol, DAG^53,54^. PKC-α in turn has been shown to trigger the phosphorylation and endocytosis of the UNC-5 receptor from the plasma membrane^55^. Therefore, higher netrin-1 concentrations induce greater PKC-α activation and UNC-5 endocytosis, and as a result, suppress the chemo-repulsive behavior. On the other hand, at lower netrin-1 concentrations, the decreased PKC-α activation may lead to an increased retention of UNC-5 in the plasma membrane, thereby promoting chemo-repulsion. Concurrently, DCC dimerization also induces protein kinase A (PKA) activation through cAMP production, which in turn has been shown to promote the translocation of DCC receptors to the plasma membrane^56^. Therefore, at higher concentrations, netrin-1-mediated DCC dimerization elicits both a positive feedback loop, which brings more DCC receptors to the plasma membrane, and a negative inhibitory loop, which removes UNC-5 receptors from the cell surface. Both pathways simultaneously promote attraction. The lower concentrations of netrin-1, on the other hand, are below a certain threshold for DCC homo-dimerization, but they are high enough to initiate a UNC5-DCC association-mediated repulsion. To directly test the role of PKC-α and PKA in the netrin-1 gradient response of hippocampal neurons, we could apply cell-permeable pharmacological inhibitors to PKC-α and PKA separately and together. Such future experiments could generate deeper insights on the integration of signaling pathways in growth cones and enable the development of a more quantitative and predictive model.

Our second major finding has been the influence of the angle of incidence of the gradient on netrin-1-mediated axon guidance. The growth cones oriented away from the gradient axis (90–135°) turned towards the source, whereas those aligned along the gradient (less than 45°) were strongly repelled (Figure 9). Micropipette-based protocols have always presented gradients at 45° to the growth cone axis, while previous microfluidic approaches have not looked at different angles of orientation. Interestingly, dynamical modeling of gradient detection in chemotactic eukaryotic cells (leukocytes) has predicted a maximal response when the gradient is incident at an angle between 40° and 80° to the axis of polarization^57^. The similarities in the signaling pathways involved in eukaryotic chemotaxis and axon guidance^58^ suggest that such an angular bias might also be relevant in growth cones. In fact, Yam et al have shown that in a Shh gradient, commissural neurons oriented at an initial angle greater than 120° made a more decisive turn towards the gradient, whereas those oriented at an initial angle less than 30° had less robust turns up the gradient^59^. Further experiments designed specifically to examine the presence of an angular bias could better elucidate this dependence. The complex interplay between the external concentrations of the chemotropic signals, the distribution of the receptors on the surface, and the graded intracellular distribution of second-messengers and effector proteins, resulting from a reaction-diffusion process, ultimately determines the response of a growth cone to a perturbation in its local environment.

Several enhancements of our microfluidic platform are possible. When we increase the number of data points (cells or chambers) we can track (or image) in a single experiment, we compromise the temporal resolution of our imaging. For example, since we were acquiring phase contrast and fluorescence images of every chamber, we could achieve a temporal resolution of approximately 20 min. More efficient image acquisition protocols that reduce the time required to image a single data point would enable us to gather migration data more frequently, which in turn might reveal interesting growth dynamics in fast-advancing cells. Additionally, our ability to trap single cells in the chambers is presently limited by Poisson statistics, but it should be possible to increase the efficiency and placement of the cells in the chamber by using microfluidic cell-trapping approaches^60,61^; integrating a single-cell trapping module to our device would enable us to utilize the full scale of the gradient generator array. In addition, more complex microfluidic control modules could be added to this platform to generate concentration gradients of one or more species, with different slopes, magnitudes, and temporal control over gradient direction.

## Conclusion

In conclusion, we have demonstrated the use of an easy-to-operate, cell-friendly, top-loadable microfluidic platform to study gradient sensing in single mammalian primary neurons. The number of available data sets per experiment is at least an order of magnitude higher than what is possible with micropipette-based assays, and it could potentially be increased by at least another factor of 5. This quantitative high-throughput single-cell approach enabled us to discern complex biphasic responses to concentrations and angles of netrin gradients. This versatile platform has the potential to greatly accelerate the discovery of complex mechanisms that govern axon guidance and cell migration by allowing the biological research community to run parallel, large-scale assays, such as stem cell differentiation in response to morphogen gradients, cell migration in cancer metastasis, wound repair or immune response.
