# Supplementary material for: Large-scale microfluidic gradient arrays reveal axon guidance behaviors in hippocampal neurons
Source: Microsyst Nanoeng. 2017 May 8;3:17003. doi: 10.1038/micronano.2017.3 (PMC6445017; doi:10.1038/micronano.2017.3)
Supplement: Supplementary Information [file micronano20173-s1.pdf]

Supplementary file

Large-scale microfluidic gradient arrays reveal axon guidance behaviors in hippocampal neurons

Nirveek Bhattacharjee and Albert Folch

Microsystems & Nanoengineering (2017) **3,** 17003; doi:10.1038/micronano.2017.3; Published online: 8 May 2017

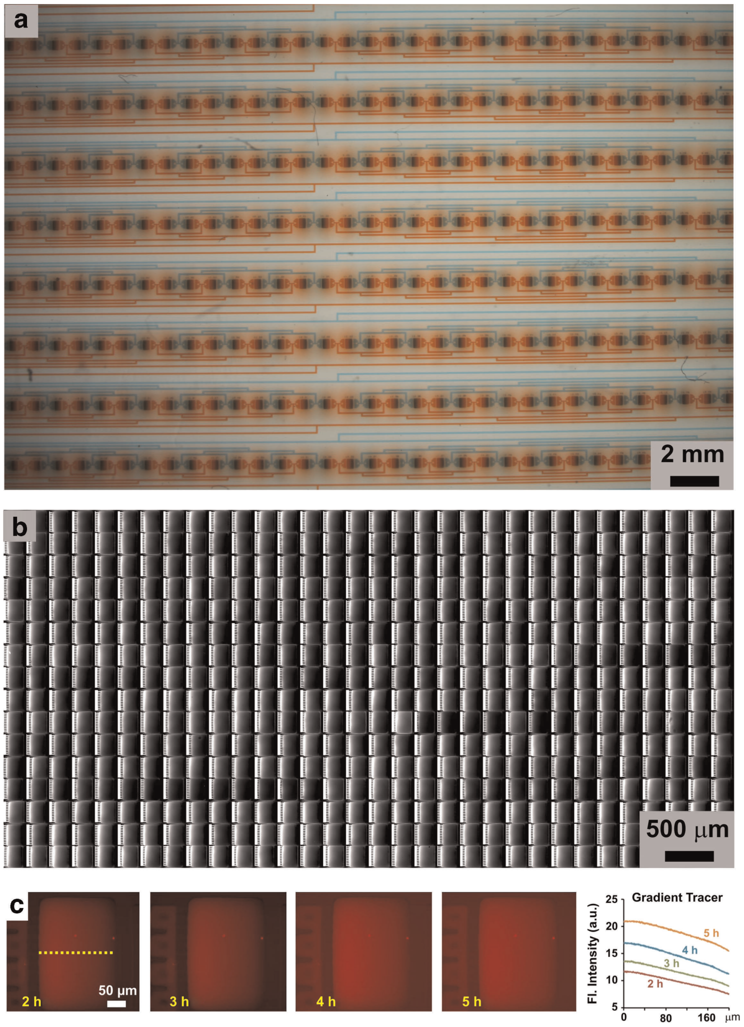

Figure S1 Large-scale gradient generator array. (**a**) A micrograph of a quarter of the array device (8 rows × 32 columns) in operation with red and blue food coloring dyes flowing from the two ends. (**b**) A large-scale montage of the surface gradient fluorescence in half of the array (16 rows and 32 columns of gradient chambers), produced by flowing 1 mM fluorescein and 45 mM Orange-G from the two ends. (**c**) Fluorescent micrograph of a chamber at the start ($t = 2$ h) of gradient application, when 3 μg mL^{-1} BSA conjugated with Texas Red was mixed in with netrin-1 as a tracer. Line-plots of the fluorescence intensity over time (2–5 h) across the width of the chamber (yellow dotted line). The gradient slope remains constant, even though the overall intensity increases with time, due to adsorption and accumulation of BSA on the surface.

Department of Bioengineering, University of Washington, 3720 15th Ave NE, Foege N423-A, Seattle, WA 98195, USA
Correspondence: Nirveek Bhattacharjee (nirveek@uw.edu)

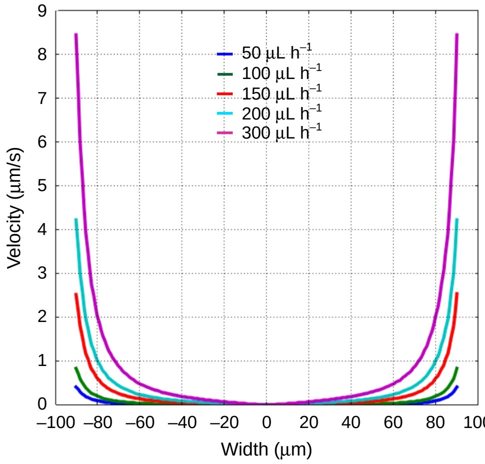

Figure S2 Finite element modeling: Flow velocity profile. A plot of the horizontal component of the fluidic velocity along the surface of the chambers for different flow rates through the device.

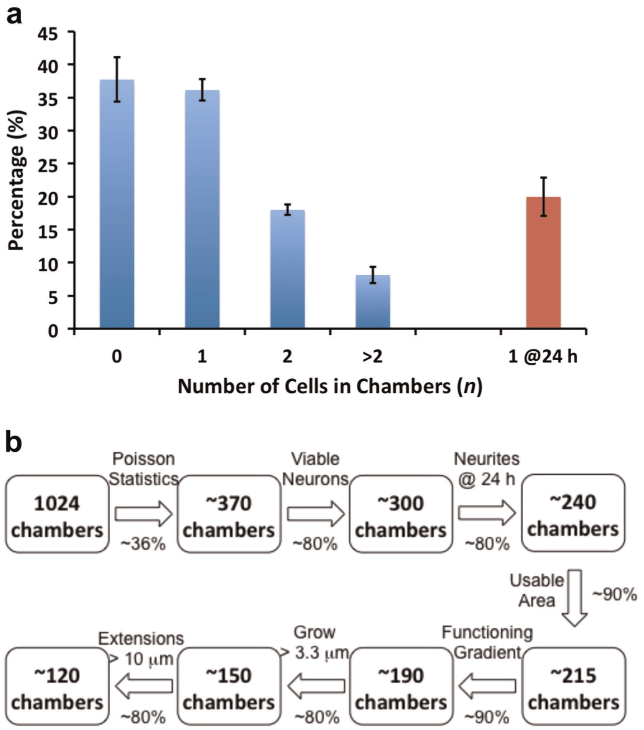

Figure S3 Cell seeding and chamber occupancy. (**a**) A bar chart showing the average percentage of chambers that had 0, 1, 2 and more than 2 neurons, right after seeding ($n=4$ experiments, blue bars). The red bar shows the percentage of chambers with neurons eligible for the experiment 24 h after seeding. (**b**) A flow-chart showing the contribution of various factors that lead to the successive decrease in yield of 'eligible' data-points per experiment.

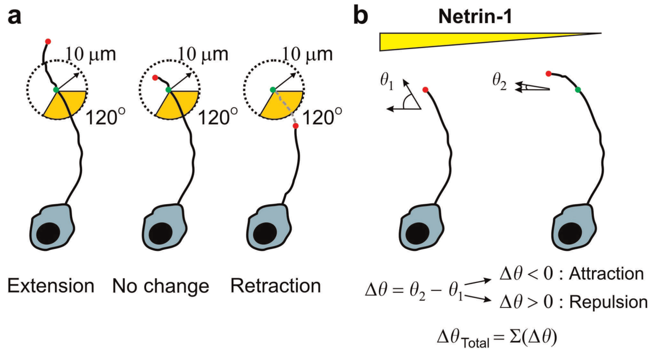

Figure S4 Schematic illustration of our algorithm for determining axon growth and turning. (**a**) Growth cone extension versus retraction, and (**b**) growth cone turning towards versus away from the gradient. The gradient vector direction is from low to high. If, for example, a growth cone is growing orthogonal to the direction of the gradient, and it turns towards the gradient, the angle between the growth cone vector and the gradient vector decreases, and therefore the change in angle is negative. The opposite is true for repulsion. Note that this definition of $\Delta\theta$ results in $\Delta\theta<0$ for chemo-attractive behavior, whereas the convention in the literature of axon guidance has been to consider attraction as $\Delta\theta>0$.

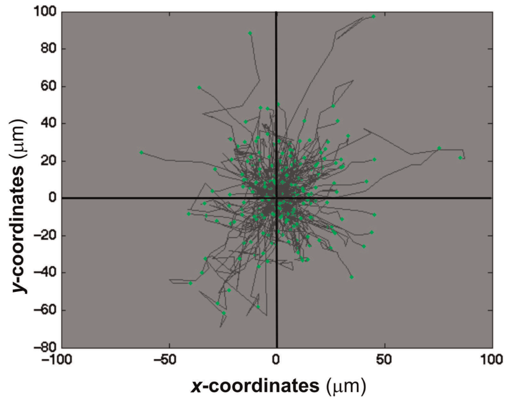

Figure S5 Growth cone trajectories. A plot of the trajectories of all the axons that were exposed to the netrin-1 gradient, and extended by at least 10 µm during the course of the experiment ($n=406$). The origin in these plots denotes the initial position of the growth cones before the start of the gradient application.

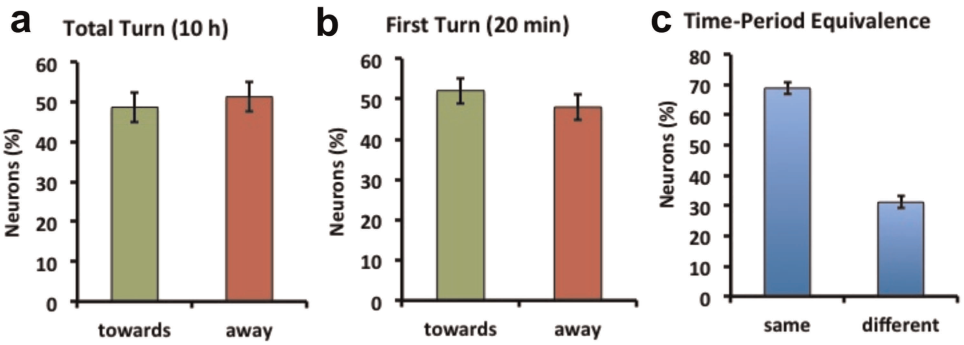

Figure S6 Towards versus away turns. (**a**) Percentage of neurons turning towards or away from the netrin-1 gradient over 10 h. (**b**) Percentage of neurons making their first turns towards or away from a netrin-1 gradient. (**c**) Percentage of neurons (with axon extensions by over 50 μm) whose overall turning behavior is in the same direction (left bar) or in a different direction (right bar) as the first turn.

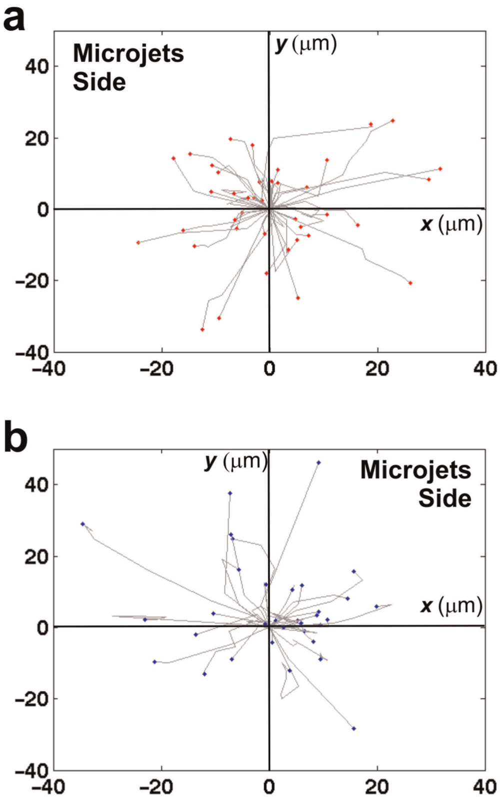

Figure S7 Growth cone trajectories in control experiments. (**a**) A plot of the trajectories of all the axons that were situated in the proximal side of the chamber (corresponding to the high-concentration quarter in the netrin-1 experiments). (**b**) A plot of the trajectories of all the axons that were situated in the distal side of the chamber (corresponding to the lowest-concentration quarter in the netrin-1 experiments). The origin in these plots denotes the initial position of the growth cones before starting the flow.

Supplementary Movie 1. Time-lapse images (taken every 30 min for 12 h) of a neuron in a chamber turning towards a gradient of netrin-1. The netrin-1 gradient (200 ng^{-1} mL^{-1}μm) starts at 2 h.

Supplementary Movie 2. Time-lapse images (taken every 30 min for 12 h) of a neuron in a chamber turning away from a gradient of netrin-1. The netrin-1 gradient (200 ng^{-1} mL^{-1} μm) starts at 2 h.

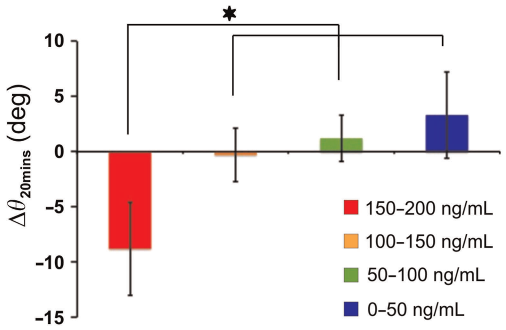

Figure S8 Concentration-dependence of the first turn after netrin-1 gradient application. Bar chart showing the average turning angle of a growth cone 20 min after being exposed to different ranges of concentration of netrin-1; all growth cones were exposed to the same linear gradient of 1 ng^{-1} $mL^{-1}$$\mu m^{-1}$. Error bars denote s.e.m. The axons exposed to 150–200 ng mL^{-1} were statistically different from the other groups (*P*-value < 0.01, Kruskal-Wallis test).

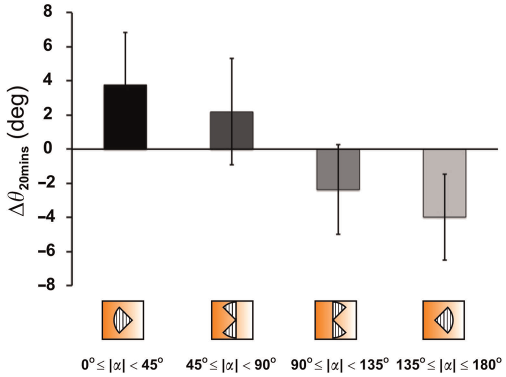

Figure S9 Angle of incidence dependence of the first turn after Netrin-1 gradient application. (**a**) Bar chart showing the mean total turning angle of a growth cone exposed to different angles of orientation (45° sectors) of the netrin-1 gradient. Black: $0° \leq |\alpha| < 45°$, Dark Gray: $45° \leq |\alpha| < 90°$, Gray: $90° \leq |\alpha| < 135°$, Light Gray: $135° \leq |\alpha| \leq 180°$.

Supplementary Movie 3. Time-lapse images (taken every 30 min for 12 h) of a neuron in a chamber extending a neurite and turning towards a gradient of netrin-1. The netrin-1 gradient (200 ng^{-1} mL^{-1} μm) starts at 2 h.
